# The interplay between acute and late toxicity among patients receiving prostate radiotherapy: an individual patient data meta-analysis of six randomised trials

**DOI:** 10.1016/S1470-2045(24)00720-4

**Published:** 2025-01-30

**Authors:** John Nikitas, Parsa Jamshidian, Alison C Tree, Emma Hall, David Dearnaley, Jeff M Michalski, W Robert Lee, Paul L Nguyen, Howard M Sandler, Charles N Catton, Himanshu R Lukka, Luca Incrocci, Wilma Heemsbergen, Floris J Pos, Soumyajit Roy, Shawn Malone, Eric Horwitz, Jessica Karen Wong, Stefano Arcangeli, Giuseppe Sanguineti, Tahmineh Romero, Yilun Sun, Michael L Steinberg, Luca F Valle, Joanne B Weidhaas, Daniel Spratt, Donatello Telesca, Amar U Kishan

**Affiliations:** **Department of Radiation Oncology** (J Nikitas MD, Prof M L Steinberg MD, L F Valle MD, Prof J B Weidhaas MD PhD, Prof A U Kishan MD)**, Department of Biostatistics** (P Jamshidian BS, D Telesca PhD)**, Department of Medicine Statistical Core** (T Romero MS)**, University of California, Los Angeles, Los Angeles, CA, USA; Institute of Cancer Research, London, UK** (A C Tree MD, E Hall PhD, Prof D Dearnaley MD)**; The Royal Marsden NHS Foundation Trust, Sutton, UK** (A C Tree, D Dearnaley)**; Department of Radiation Oncology, Washington University School of Medicine, St. Louis, MO, USA** (Prof J M Michalski MD)**; Department of Radiation Oncology, Duke University, Durham, NC, USA** (Prof W R Lee MD)**; Department of Radiation Oncology, Dana-Farber Cancer Institute and Brigham and Women’s Hospital, Boston, MA, USA** (Prof P L Nguyen MD)**; Department of Radiation Oncology, Cedars-Sinai Medical Center, Los Angeles, CA, USA** (Prof H M Sandler MD)**; Department of Radiation Oncology, Princess Margaret Cancer Centre and University of Toronto, Toronto, ON, Canada** (Prof C N Catton MD)**; Juravinski Cancer Centre at Hamilton Health Sciences, Hamilton, ON, Canada** (Prof H R Lukka MD)**; Department of Radiotherapy, Erasmus Medical Center, Rotterdam, Netherlands** (Prof L Incrocci MD PhD, W Heemsbergen PhD)**; Department of Radiation Oncology, The Netherlands Cancer Institute, Amsterdam, Netherlands** (F J Pos MD PhD)**; Department of Radiation Oncology, Rush University Medical Center, Chicago, IL, USA** (S Roy MD)**; Department of Radiology, Radiation Oncology and Medical Physics, University of Ottawa, Ottawa, ON, Canada** (Prof S Malone MD)**; Department of Radiation Oncology, Fox Chase Cancer Center, Philadelphia, PA, USA** (Prof E Horwitz MD, J K Wong MD)**; Department of Medicine and Surgery, University of Milan Bicocca, Milan, Italy** (S Arcangeli MD)**; Department of Radiation Oncology, IRCCS Regina Elena National Cancer Institute, Rome, Italy** (Prof G Sanguineti MD)**; Department of Radiation Oncology, University Hospitals Seidman Cancer Center, Case Western Reserve University School of Medicine, Cleveland, OH, USA** (Y Sun PhD, Prof D Spratt MD)**; Greater Los Angeles VA Medical Center, Los Angeles, CA, USA** (L F Valle)**; Department of Radiation Oncology, University Hospitals Seidman Cancer Center, Case Western Reserve University, Cleveland, OH, USA** (S Roy)**; Department of Radiation Oncology, University of Pennsylvania, Philadelphia, PA, USA** (J Nikitas)

## Abstract

**Background:**

The association between acute and late toxicity following prostate radiotherapy has not been well studied using data from multiple randomised clinical trials and fractionation schedules. We aimed to characterise the relationship between acute and late genitourinary and gastrointestinal toxicity among patients receiving conventionally fractionated or moderately hypofractionated prostate radiotherapy.

**Methods:**

This was an individual patient data meta-analysis that identified randomised phase 3 trials of conventionally fractionated or moderately hypofractionated prostate radiotherapy in the Meta-Analysis of Randomized trials in Cancer of the Prostate (MARCAP) Consortium that had individual-level acute and late toxicity data available and were available before Dec 1, 2023. Trials without individual patient data were excluded. Data were provided to MARCAP by study investigators. The associations between acute (≤3 months after radiotherapy) and late (>3 months after radiotherapy) grade 2 or greater genitourinary and gastrointestinal toxicities were assessed using adjusted generalised linear mixed models (adjusted for age, androgen deprivation therapy status, type of radiotherapy, radiation dose, and radiation schedule). In the subset of trials that collected Expanded Prostate Cancer Index Composite quality of life (QOL) evaluations, the association between acute genitourinary and gastrointestinal toxicity and decrements at least twice the minimal clinically important difference (MCID) for urinary and bowel QOL were also evaluated.

**Findings:**

Six of 26 available trials met all the eligibility criteria. 6593 patients were included (conventionally fractionated: n=4248; moderately hypofractionated: n=2345). Median follow-up was 72 months (IQR 61–94). Acute grade 2 or greater genitourinary toxicity was associated with both late grade 2 or greater genitourinary toxicity (odds ratio 2·20 [95% CI 1·88–2·57], p<0·0001) and decrement at least twice the MCID in urinary QOL (1·41 [1·17–1·68], p=0·0002). Acute grade 2 or greater gastrointestinal toxicity was associated with both late grade 2 or greater gastrointestinal toxicity (2·53 [2·07–3·08], p<0·0001) and decrement at least twice the MCID in bowel QOL (1·52 [1·26–1·83], p<0·0001).

**Interpretation:**

Acute toxicity following prostate radiotherapy was statistically significantly associated with late toxicity and with decrement in patient-reported QOL metrics. These data support efforts to evaluate whether interventions that reduce acute toxicity ultimately reduce the risk of late toxicity.

## Introduction

Dose-escalated external beam radiotherapy is the standard of care for localised prostate cancer with excellent biochemical control rates.^1–5^ Toxicity following radiotherapy is thought to reflect direct cellular injury or death from radiation, bystander effects in unirradiated cells, and immune reactions to radiation, including inflammation.^6^ Acute toxicity after radiotherapy, operationally defined as occurring within 3 months of the completion of treatment, is thought to reflect the death of rapidly proliferating cells and is generally considered reversible. By contrast, late toxicity is typically thought to reflect fibrosis and chronic inflammation, and is generally considered to be more detrimental to patient quality of life (QOL).^7–9^

Although both forms of toxicity clearly follow from the irradiation of normal tissues during the delivery of therapeutic radiation, the association between the two has not been well studied using robust data from multiple randomised clinical trials or fractionation schedules. Given the long survival of patients with prostate cancer, late toxicity following any form of treatment, including radiotherapy, remains paramount in shared decision making.^10,11^ If a link between acute and late toxicity were to exist, this could have several important implications. First, this might provide an opportunity to intervene in patients experiencing acute toxicity to mitigate further late toxicity. Second, this could inform the overall impact of interventions targeting acute toxicity, as these might in turn have an effect on the more important endpoint of late toxicity as well.

Herein, we sought to characterise the relationship between acute physician-scored genitourinary and gastrointestinal toxicity following prostate radiotherapy and both late physician-scored genitourinary and gastrointestinal toxicity as well as a clinically significant decrement in patient-reported urinary and bowel QOL. To maximise data fidelity and quality, we leveraged individual patient data from patients enrolled on randomised trials with long-term follow-up. Since the initial randomised trials were conducted to analyse other endpoints and not the association between acute and late toxicity, we conducted an individual patient data meta-analysis to study the association between acute and late toxicity across different radiotherapy regimens, androgen deprivation therapy regimens, and patient populations. These trials all reflected representative patient populations who were treated in the contemporary setting with definitive prostate radiotherapy. Importantly, all patients were treated and followed with prospective collection of toxicity data.

## Methods

### Search strategy and selection criteria

In this individual patient data meta-analysis, we identified prospective randomised trials in the Meta-Analysis of Randomized trials in Cancer of the Prostate (MARCAP) Consortium that included men who received conventionally fractionated or moderately hypo-fractionated radiation therapy for prostate cancer, had individual patient data available regarding treatment-related acute and late toxicity, and were available before beginning this analysis on Dec 1, 2023. The MARCAP Consortium has been previously described.^12^ Briefly, it contains individual patient data from randomised clinical trials run through multiple collaborative groups (including NRG/RTOG, Institute of Cancer Research, Ontario Clinical Oncology Group, and Dutch Cancer Society) as well as individual institutional trials that have been shared by the study investigators. Studies without individual patient data available for acute and late toxicity were excluded.

### Data analysis

A statistical plan was created before individual patient data pooling and analysis. All analyses were performed on an intention-to-treat basis. Acute and late toxicity events were defined as per the original trial definitions (table 1). Irrespective of the toxicity grading system used, acute toxicity was defined as occurring up to 3 months from treatment completion and late toxicity was defined as occurring more than 3 months from treatment completion. Time to toxicity data were not uniformly available, thus toxicity grade 2 or greater, defined as a binary event per person (ever occurring versus never occurring), was the primary endpoint. Patient-reported urinary and bowel QOL data, as assessed using the Expanded Prostate Cancer Index Composite (EPIC) questionnaire, were analysed to identify instances of decrement at least twice the minimal clinically important difference (MCID) at any time more than 3 months from treatment completion during a 60-month follow-up period. The MCID threshold was defined as half the across-trial standard deviation of the baseline EPIC score in that given domain, on a per-trial basis.^13^ MCID represents the smallest clinically noticeable difference and at least twice the MCID represents the smallest clinically significant difference.

The relationship between acute and late toxicity was evaluated in a cross-trial meta-analysis using the following two forms of late toxicity endpoints: binary late grade 2 or greater toxicity and binary twice (or more) the MCID QOL decrement. An odds ratio was reported with a 95% CI. The effect of acute toxicity on the binary late grade 2 or greater toxicity outcome was assessed using a generalised linear mixed model with a trial-specific random intercept, adjusting for potential patient and treatment-related confounders. Models were adjusted for age (continuous variable), androgen deprivation therapy (ADT) status (yes or no), type of radiotherapy (three-dimensional conformal radiotherapy or intensity-modulated radiotherapy), radiation dose (<74 Gy, 74–80 Gy, or >80 Gy; converted to an equivalent dose in 2 Gy fractions [EQD2], adjusted for the effect on late-responding tissue using an α/β ratio of 3), and radiation schedule (conventionally fractionated or moderately hypofractionated) as fixed effects. These were not adjusted for the competing risk of death. Cumulative incidence rates of late grade 2 or greater genitourinary and gastrointestinal toxicity were calculated using data from the four clinical trials with time-to-toxicity data available. These were stratified by acute toxicity status (grade <2 or grade ≥2) and adjusted for the competing risk of death. The same generalised linear mixed models and covariates were used to evaluate the binary twice (or more) the MCID urinary and bowel QOL decrement endpoints. These were derived from longitudinally measured patient-reported EPIC scores for urinary and bowel QOL. For evaluation of QOL metrics, missing baseline scores were imputed using the predicted EPIC baseline score from a linear mixed model, where time was a covariate alongside a nested patient-trial random effect. Interactions between acute toxicity and trial group were considered in all models and dropped if statistically insignificant. Intertrial heterogeneity was evaluated for differences in the effect size of acute grade 2 or greater genitourinary or gastrointestinal toxicity on late grade 2 or greater genitourinary or gastrointestinal toxicity or decrements at least twice the MCID for urinary or bowel QOL using *I*^2^ and Cochran’s Q. Sensitivity analyses were performed to evaluate the association between acute grade 2 or greater genitourinary and gastrointestinal toxicity and late grade 3 or greater genitourinary and gastrointestinal toxicity as well as the association between acute grade 2 or greater toxicity and decrement at least twice the MCID at 2 years, at the time of last follow-up, or when excluding patients whose missing baseline QOL scores had to be imputed. We used five-fold cross-validation to calculate the average area under the curve (AUC), sensitivity, and specificity for the generalised linear mixed models predicting late grade 2 or greater genitourinary toxicity, late grade 2 or greater gastrointestinal toxicity, decrement at least twice the MCID in urinary QOL, and decrement at least twice the MCID in bowel QOL. All statistical analyses were conducted using R (version 4.4.0), specifically employing the lme4,^14^ glmmTMB,^15^ survival,^16^ and cmprsk^17^ packages.

### Role of the funding source

The funders of this study had no role in the study design, data collection, data analysis, data interpretation, or writing of the report.

## Results

**Figure 1:**
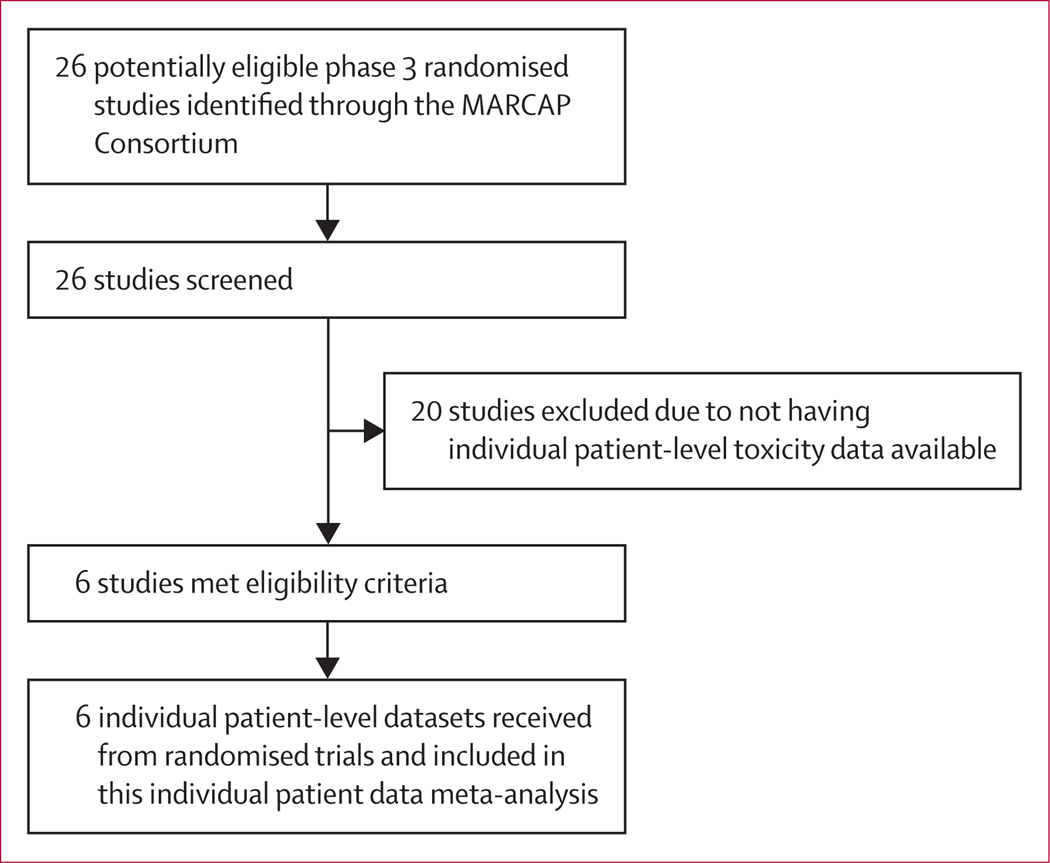
Study selection Full details on included trials are provided in table 1. MARCAP=Meta-Analysis of Randomized trials in Cancer of the Prostate.

Our search results identified 26 phase 3 randomised controlled trials through the MARCAP consortium, six of which met all eligibility criteria (figure 1): CHHiP, RTOG 0126, PROFIT, RTOG 0415, Ottawa 0101, and Fox Chase Cancer Center, and included data from 6593 patients.

CHHiP (Conventional or Hypofractionated High-dose Intensity Modulated Radiotherapy in Prostate Cancer; CRUK/06/016) was a trial comparing conventionally fractionated radiotherapy (74 Gy in 37 fractions) versus one of two moderately hypo fractionated radiotherapy schedules (60 Gy in 20 fractions or 57 Gy in 19 fractions).^18^ RTOG 0126 was a randomised trial comparing standard (70·2 Gy in 39 fractions) and dose-escalated (79·2 Gy in 44 fractions) conventionally fractionated radiotherapy.^2^ PROFIT was a multicentre, randomised, noninferiority trial comparing conventionally fractionated radiotherapy (78 Gy in 39 fractions) and moderately hypofractionated radiotherapy (60 Gy in 20 fractions).^19^ RTOG 0415 was a randomised trial comparing conventionally fractionated radiotherapy (73·8 Gy in 41 fractions) and moderately hypofractionated radiotherapy (70 Gy in 28 fractions).^3,4^ Ottawa 0101 was a randomised trial comparing neoadjuvant and concurrent ADT for 6 months versus concurrent and adjuvant ADT for 6 months.^20^ Both treatment groups received conventionally fractionated radiotherapy (76 Gy in 38 fractions). The Fox Chase Cancer Center trial compared conventionally fractionated radiotherapy (76 Gy in 38 fractions) versus moderately hypofractionated radiotherapy (70·2 Gy in 26 fractions; table 1, appendix p 2).^5,21^

Median follow-up was 72 months (IQR 61–94). 6101 (92·5%) patients had acute toxicity data available, all 6593 patients had late toxicity data available, 3705 (56·2%) patients had time-to-late toxicity data available, and 3559 (54·0%) patients had EPIC QOL data available. EPIC QOL data were only available for the RTOG 0415, PROFIT, and CHHiP trials and baseline scores were missing for approximately 45% of patients in both the urinary and bowel functional domains.

The median age of patients across the six trials was 69 years (IQR 64–74; table 2). 4248 (64·4%) of 6593 patients were treated with conventionally fractionated radiotherapy, and 2345 (35·6%) of 6593 patients were treated with moderately hypofractionated radiotherapy. This imbalance occurred because some trials did not randomise between conventionally fractionated and moderately hypofractionated radiotherapy. 4939 (74·9%) of 6593 patients were treated using intensity-modulated radiotherapy. Median EQD2 was 74 Gy (IQR 72–76) and 2670 (40·5%) of 6593 patients received ADT.

Acute grade 2 or greater genitourinary toxicity was reported in 1817 (29·8%) of 6101 patients and late grade 2 or greater genitourinary toxicity was reported in 1017 (15·4%) of 6593 patients (table 3). Acute grade 2 or greater gastrointestinal toxicity was reported in 897 (14·7%) of 6101 patients and late grade 2 or greater gastrointestinal toxicity was reported in 941 (14·3%) of 6593 patients. Among patients that had time-to-late toxicity data available, more than 95% of late grade 2 or greater genitourinary and gastrointestinal toxicities were first reported beyond 6 months post-treatment (635 [95·2%] of 667 and 693 [95·5%] of 726, respectively).

**Figure 2:**
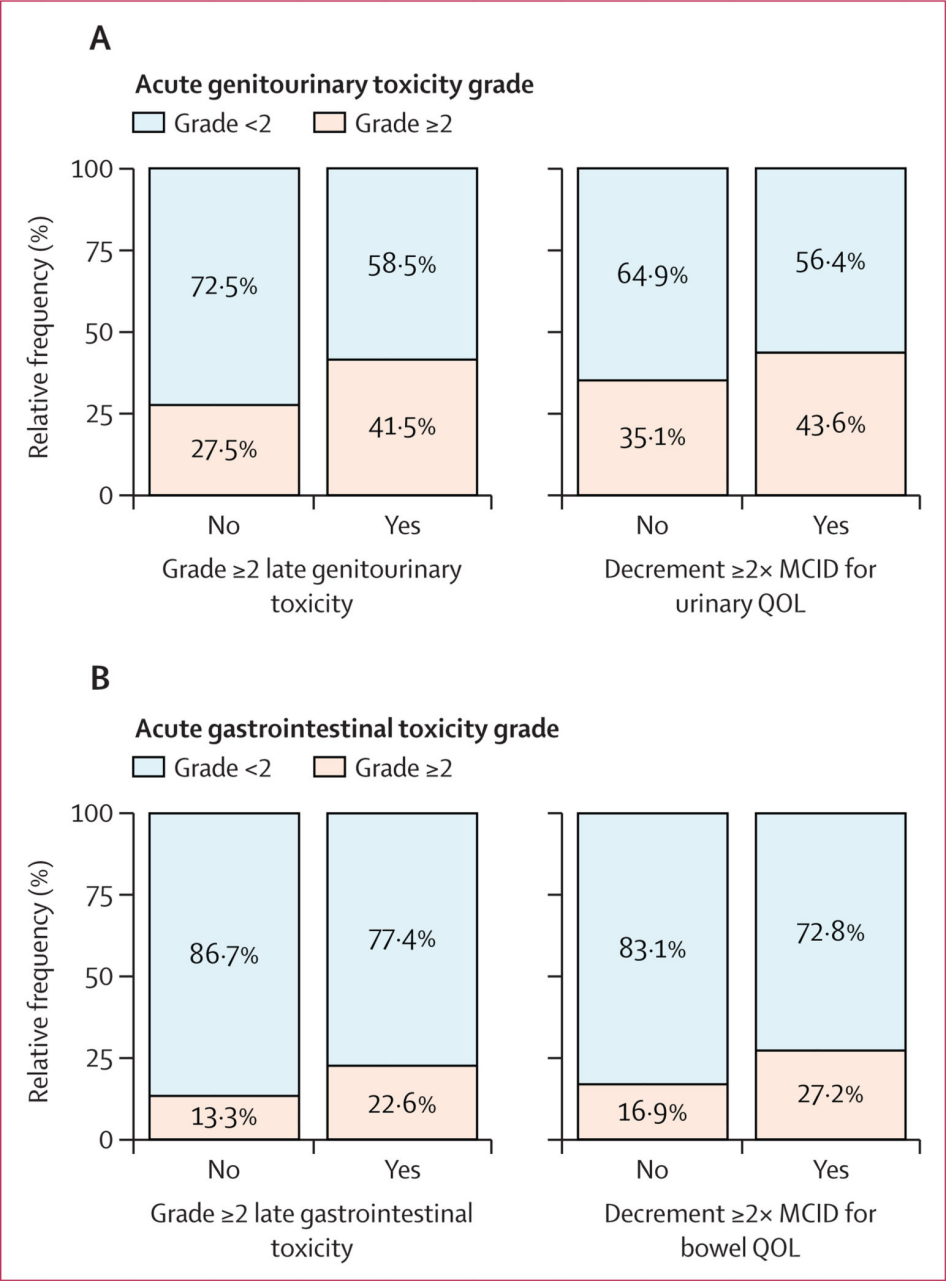
Relative frequency of grade ≥2 genitourinary toxicity (A) and grade ≥2 gastrointestinal toxicity (B) Data are for patients with or without late grade ≥2 genitourinary toxicity or decrement ≥2× MCID in urinary QOL (A) and among patients with or without late grade ≥2 gastrointestinal toxicity or decrement ≥2× MCID in bowel QOL (B). MCID=minimal clinically important difference. QOL=quality of life.

**Figure 3:**
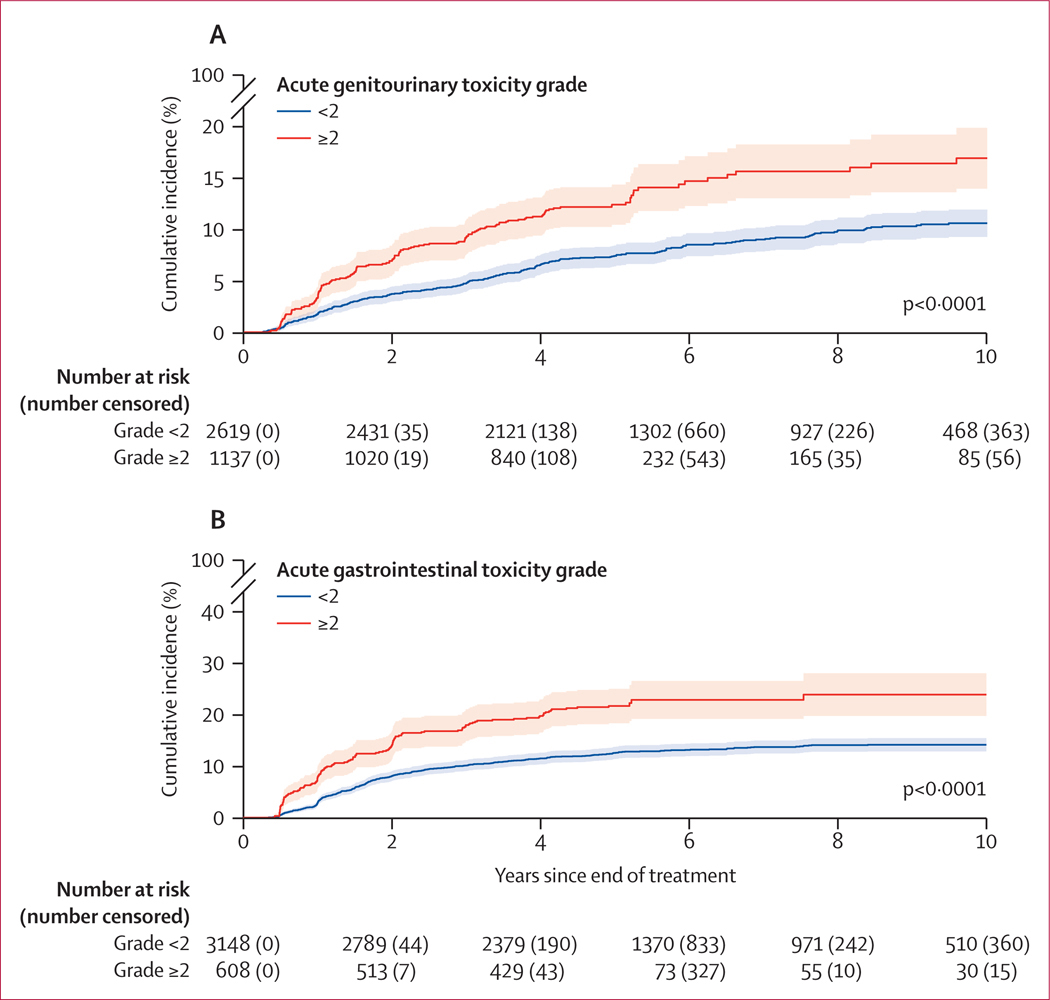
Cumulative incidence curves for late grade ≥2 genitourinary toxicity (A) and late grade ≥2 gastrointestinal toxicity (B) Data are for patients with or without acute grade ≥2 genitourinary toxicity (A) and for patients with or without acute grade ≥2 gastrointestinal toxicity (B). The shaded areas represent 95% CIs.

Acute grade 2 or greater genitourinary toxicity was associated with late grade 2 or greater genitourinary toxicity (odds ratio [OR] 2·20 [95% CI 1·88–2·57], p<0·0001; figure 2A, appendix p 9). At 5 years from the end of treatment, the cumulative incidence of late grade 2 or greater genitourinary toxicity was 12·5% (95% CI 10·5–14·5) for patients with acute grade 2 or greater genitourinary toxicity and 7·5% (6·4–8·5) for patients without acute grade 2 or greater genitourinary toxicity (p<0·0001, figure 3A). Acute grade 2 or greater genitourinary toxicity was not associated with radiation dose, intensity-modulated radiation therapy use, ADT use, or use of a moderately hypofractionated regimen (data not shown). The odds of experiencing late grade 2 or greater genitourinary toxicity were statistically significantly higher with moderately hypofractionated compared with conventionally fractionated radiotherapy (OR 1·37 [95% CI 1·15–1·64], p=0·0005). Additionally, late grade 2 or greater genitourinary toxicity was statistically significantly associated with radiation dose levels. Specifically, compared with EQD2 less than 74 Gy, the odds were statistically significantly higher for EQD2 74–80 Gy (OR 1·20 [95% CI 1·03–1·41], p=0·021) but not for EQD2 greater than 80 Gy (1·08 [0·60–1·94], p=0·79).

Acute grade 2 or greater gastrointestinal toxicity was statistically significantly associated with late grade 2 or greater gastrointestinal toxicity (OR 2·53 [95% CI 2·07–3·08], p<0·0001; figure 2B, appendix p 10). At 5 years, the cumulative incidence of late grade 2 or greater gastrointestinal toxicity was 21·5% (95% CI 18·2–24·8) for patients with acute grade 2 or greater gastrointestinal toxicity and 12·5% (11·3–13·6) for patients without acute grade 2 or greater gastrointestinal toxicity (p<0·0001; figure 3B). Acute grade 2 or greater gastrointestinal toxicity was statistically significantly associated with moderately hypofractionated radiotherapy (OR 1·49 [95% CI 1·21–1·83], p=0·0002). Late grade 2 or greater gastrointestinal toxicity was statistically significantly associated with radiation dose. Specifically, compared with EQD2 less than 74 Gy, the odds were higher for EQD2 74–80 Gy (OR 1·46 [95% CI 1·24–1·71], p<0·0001) but not for EQD2 greater than 80 Gy (0·96 [0·46–2·02], p=0·92).

693 (19·5%) of 3548 patients experienced decrement of at least twice the MCID in urinary QOL and 1109 (31·3%) of 3539 patients experienced decrement of at least twice the MCID in bowel QOL.

Acute grade 2 or greater genitourinary toxicity was associated with decrement at least twice the MCID in urinary QOL (OR 1·41 [95% CI 1·17–1·68], p=0·0002; figure 2A, appendix p 11). Among patients who experienced decrement at least twice the MCID, 46 (21·1%) of 218 recovered to baseline after a median of 30 months (IQR 20–48), 30 (13·8%) of 218 recovered to the MCID after a median of 12 months (IQR 12–24), and 142 (65·1%) of 218 did not recover.

Acute grade 2 or greater gastrointestinal toxicity was associated with decrement at least twice the MCID in bowel QOL (OR 1·52 [95% CI 1·26–1·83], p<0·0001; figure 2B, appendix p 12). Among patients who experienced decrement at least twice the MCID, 158 (32·0%) of 494 recovered to baseline after a median of 24 months (IQR 18–48), 54 (10·9%) of 494 recovered to the MCID after a median of 12 months (IQR 6–24 months), and 282 (57·1%) of 494 did not recover.

Assessments of inter-trial heterogeneity are shown in the appendix (p 3). There was statistically significant inter-trial heterogeneity for the effect size of acute grade 2 or greater genitourinary toxicity on decrement at least twice the MCID in urinary QOL (*I*^2^=79%, p=0·0085). There was no significant inter-trial heterogeneity for the effect size of acute grade 2 or greater genitourinary toxicity on late grade 2 or greater genitourinary toxicity or the effect size of acute grade 2 or greater gastrointestinal toxicity on late grade 2 or greater gastrointestinal toxicity or decrement at least twice the MCID in bowel QOL.

Sensitivity analyses showed similar findings when examining the association between acute grade 2 or greater genitourinary and gastrointestinal toxicity and late grade 3 or greater genitourinary and gastrointestinal toxicity (appendix p 4). Sensitivity analyses showed similar findings when examining only QOL decrements present at 2 years or at the time of last follow-up, or when excluding patients whose missing baseline QOL scores had to be imputed (appendix pp 5–7). For each model, sensitivity values ranged between 56% and 63%, specificity between 56% and 63%, and AUC values between 0·57 and 0·69 (appendix p 8).

## Discussion

In this individual patient data meta-analysis of six randomised controlled trials, we found that there was a statistically significant association between acute moderate or worse physician-scored genitourinary and gastrointestinal toxicity and late moderate or worse both physician-scored genitourinary and gastrointestinal toxicity and patient-reported clinically significant decrements in urinary and bowel QOL. To our knowledge, this provides the best evidence to date supporting the phenomenon of consequential late toxicity following radiotherapy in the context of prostate cancer^22^ and represents one of the largest analyses so far with inclusion of both standard and dose-escalated conventionally fractionated radiotherapy regimens as well as dose-escalated moderately hypofractionated regimens. This supports the need for further prospective studies to evaluate whether strategies that mitigate the risk of acute toxicity translate into reduced rates of late toxicity.

Interventions that have been shown to reduce acute toxicity following radiotherapy, which include aggressive margin reduction with MRI-guidance, urethral-sparing techniques, and placement of a perirectal spacer, are thought to do so by modifying the physical dose distribution experienced by normal tissues at the time of radiotherapy delivery.^23–25^ The implications for late toxicity are less clear. This is intuitive, as late toxicity is thought to reflect multiple complex and overlapping pathophysiological processes, including inflammation and fibrosis, whereas acute toxicity likely reflects cell death induced at the time of radiation. The present study, which supports the occurrence of consequential late effects, suggests that reducing acute toxicity could ultimately reduce late toxicity, though the associations in our study are relatively modest (ORs of 1·4–2·5 and AUCs of 0·57–0·69) and cannot be used to infer causation. That said, mitigating late toxicity without mitigating acute toxicity could be challenging, given that this might require modifying these other biological pathways or increasing our understanding of each patient’s intrinsic late response to radiation, which might be dependent on their germline DNA.^26^

The current results are consistent with previously reported analyses of either individual clinical trials or single institution retrospective cohorts that supported an association between acute and late toxicity in the setting of conventionally fractionated radiation therapy.^27–30^ A post-hoc analysis of the PACE-B trial reported similar findings in the setting of ultra-hypofractionated radiation therapy.^31^ The present results extend these previous studies by including data from over 6000 patients treated across multiple multicentre randomised trials with rigorous follow-up and diverse radiotherapy fractionation schedules, doses, and planning techniques, and including the correlation between acute toxicity and late decrements in patient-reported outcomes as well.

This study had several limitations. First, these are post-hoc analyses of six different randomised controlled trials, so there could be unmeasured confounders influencing our observations. Second, we did not have enough data on patients’ medical comorbidities to adjust for them in our analysis. Third, trial toxicity data relied on physician or investigator reporting, which might not capture all events. Fourth, we only had data about the worst physician-scored toxicity noted for each patient and not on the resolution or temporal evolution of physician-scored toxicity. Finally, three of the trials did not collect longitudinal EPIC data for the QOL analysis and the trials that did collect these data had missing baseline information for approximately 45% of patients. This missing QOL data could have introduced a source of bias in our analysis.

In conclusion, acute toxicity following prostate radiotherapy was statistically significantly associated with late toxicity and late decrement in patient-reported QOL metrics following conventionally fractionated or moderately hypofractionated prostate radiotherapy. Further prospective studies are needed to evaluate whether strategies that mitigate the risk of acute toxicity lead to reduced rates of late toxicity and whether early interventions to treat acute toxicity impact rates of late toxicity.

## Supplementary Material

Supplementary

## Figures and Tables

**Table 1:** Trials included in individual patient data meta-analysis

	Inclusion criteria	Study period	Intervention group	Control group	Primary endpoint	Toxicity scoring	QOL evaluation

**Iso-effective dose trials**							
CHHiP	T1b–T3a, PSA <40 ng/mL	2002–2011	n=1043; 60 Gy in 3 Gy fractions*	n=1050; 74 Gy in 2 Gy fractions	Time to biochemical or clinical failure	RTOG	EPIC
PROFIT	T1–T2a, Gleason score ≤6, and PSA 10·1–20 ng/mL; T2b–c, Gleason score ≤6, and PSA ≤20 ng/mL; or T1–2, Gleason score 7, and PSA ≤20 ng/mL	2006–2011	n=608; 60 Gy in 3 Gy fractions	n=598; 78 Gy in 2 Gy fractions	Biochemical clinical failure	RTOG	EPIC
**Dose-escalated trials**							
RTOG 0126	T1b–T2b, Gleason score ≤6 and PSA <20 ng/mL or Gleason score 7 and PSA <15 ng/mL	2002–2008	n=739; 79·2 Gy in 1·8 Gy fractions	n=746; 70·2 Gy in 1·8 Gy fractions	Overall survival	CTCAE version 2.0 (acute) and RTOG/EORTC (late)	NA
RTOG 0415	T1–T2a, Gleason score ≤6, and PSA <10 ng/mL	2006–2009	n=545; 70 Gy in 2·5 Gy fractions	n=534; 73·8 Gy in 1·8 Gy fractions	Disease-free survival	CTCAE version 3.0	EPIC
FCCC	Gleason score 7, PSA 10–20, or ≥3 biopsy cores with Gleason scores ≥6 or Gleason score 8–10 or Gleason score 7 in ≥4 cores, cT3, or PSA>20 ng/mL	2002–2006	n=149; 70·2 Gy in 2·7 Gy fractions	n=149; 76 Gy in 2 Gy fractions	Biochemical or clinical disease failure	RTOG/LENT-SOMA	NA
**Androgen deprivation therapy trials**							
Ottawa 0101	T1b-T3a, Gleason score ≤7 and PSA <30 ng/mL	2002–2012	n=215; 76 Gy in 2 Gy fractions with neoadjuvant and concurrent ADT	n=217; 76 Gy in 2 Gy fractions with concurrent and adjuvant ADT	Biochemical relapse-free survival	RTOG (acute) and RTOG/EORTC (late)	NA

n=number of patients included in the analysis from each clinical trial group. ADT=androgen deprivation therapy. CHHiP=conventional or hypofractionated high-dose intensity-modulated radiotherapy for prostate cancer. CTCAE=Common Terminology Criteria for Adverse Events. EORTC=European Organisation for Research and Treatment of Cancer. EPIC=Expanded Prostate Cancer Index Composite. FCCC=Fox Chase Cancer Center. LENT-SOMA=Late Effects Normal Tissue Task Force-Subjective, Objective, Management, Analytic. NA=not applicable. PROFIT=Prostate Fractionated Irradiation Trial. PSA=prostate-specific antigen. QOL=quality of life. RTOG=Radiation Therapy Oncology Group.

* Or 57 Gy in 19 fractions, not included in this meta-analysis.

**Table 2:** Patient and treatment characteristics

	CHHiP (n=2093)	RTOG 0126 (n=1485)	PROFIT (n=1206)	RTOG 0415 (n=1079)	Ottawa 0101 (n=432)	FCCC (n=298)	Overall (n=6593)

Age, years	69 (65–73)	71 (65–74)	71 (67–75)	67 (62–72)	70 (66–74)	68 (62–73)	69 (64–74)
ECOG performance status							
Unknown	2093 (100%)	··	64 (5·3%)	··	14 (3·2%)	··	2171 (32·9%)
0	··	1358 (91·4%)	1048 (86·9%)	998 (92·5%)	282 (65·3%)	282 (94·6%)	3968 (60·2%)
1	··	127 (8·6%)	90 (7·5%)	81 (7·5%)	134 (31·0%)	15 (5·0%)	447 (6·8%)
2	··	··	3 (0·2%)	··	2 (0·5%)	1 (0·3%)	6 (0·1%)
3	··	··	1 (0·1%)	··	··	··	1 (<0·1%)
Radiotherapy modality							
3D-CRT	··	985 (66·3%)	12 (1·0%)	225 (20·9%)	432 (100%)	··	1654 (25·1%)
IMRT	2093 (100%)	500 (33·7%)	1194 (99·0%)	854 (79·1%)	··	298 (100%)	4939 (74·9%)
Fraction							
CF	1050 (50·2%)	1485 (100%)	598 (49·6%)	534 (49·5%)	432 (100%)	149 (50·0%)	4248 (64·4%)
MHF	1043 (49·8%)	··	608 (50·4%)	545 (50·5%)	··	149 (50·0%)	2345 (35·6%)
Radiation dose (EQD2*)							
<74 Gy	1056 (50·5%)	746 (50·2%)	608 (50·4%)	534 (49·5%)	··	1 (0·3%)	2945 (44·7%)
74–80 Gy	1037 (49·5%)	739 (49·8%)	598 (49·6%)	545 (50·5%)	432 (100%)	148 (49·7%)	3499 (53·1%)
>80 Gy	··	··	··	··	··	149 (50·0%)	149 (2·3%)
ADT							
Unknown	7 (0·3%)	··	··	··	··	··	7 (0·1%)
Yes	2032 (97·1%)	··	68 (5·6%)	··	432 (100%)	138 (46·3%)	2670 (40·5%)
No	54 (2·6%)	1485 (100%)	1138 (94·4%)	1079 (100%)	··	160 (53·7%)	3916 (59·4%)

Data are median (IQR) or n (%). 3D-CRT=three-dimensional conformal radiation therapy. ADT=androgen deprivation therapy. CF=conventional fractionation.

CHHiP=conventional or hypofractionated high dose intensity modulated radiotherapy for prostate cancer. ECOG=Eastern Cooperative Oncology Group. EQD2=equivalent dose in 2 Gy fractions. FCCC=Fox Chase Cancer Center. HYPRO=hypofractionated irradiation for prostate cancer. IMRT=intensity-modulated radiation therapy.

MHF=moderate hypofractionation. PROFIT=Prostate Fractionated Irradiation Trial. RTOG=Radiation Therapy Oncology Group.

* EQD2 calculated using α/β=3.

**Table 3:** Acute and late toxicity rates

	CHHiP	RTOG 0126	PROFIT	RTOG 0415	Ottawa 0101	FCCC	Overall

**Acute GU toxicity**							
Grade <2	842/1625 (51·8%)	1212/1461 (83·0%)	838/1206 (69·5%)	787/1079 (72·9%)	361/432 (83·6%)	244/298 (81·9%)	4284/6101 (70·2%)
Grade ≥2	783/1625 (48·2%)	249/1461 (17·0%)	368/1206 (30·5%)	292/1079 (27·1%)	71/432 (16·4%)	54/298 (18·1%)	1817/6101 (29·8%)
**Acute GI toxicity**							
Grade <2	1125/1625 (69·2%)	1375/1461 (94·1%)	1039/1206 (86·2%)	966/1079 (89·5%)	411/432 (95·1%)	288/298 (96·6%)	5204/6101 (85·3%)
Grade ≥2	500/1625 (30·8%)	86/1461 (5·9%)	167/1206 (13·8%)	113/1079 (10·5%)	21/432 (4·9%)	10/298 (3·4%)	897/6101 (14·7%)
**Late GU toxicity**							
Grade <2	1938/2093 (92·6%)	1315/1485 (88·6%)	917/1206 (76·0%)	797/1079 (73·9%)	377/432 (87·3%)	232/298 (77·9%)	5576/6593 (84·6%)
Grade ≥2	155/2093 (7·4%)	170/1485 (11·4%)	289/1206 (24·0%)	282/1079 (26·1%)	55/432 (12·7%)	66/298 (22·1%)	1017/6593 (15·4%)
**Late GI toxicity**							
Grade <2	1874/2093 (89·5%)	1210/1485 (81·5%)	1065/1206 (88·3%)	883/1079 (81·8%)	349/432 (80·8%)	271/298 (90·9%)	5652/6593 (85·7%)
Grade ≥2	219/2093 (10·5%)	275/1485 (18·5%)	141/1206 (11·7%)	196/1079 (18·2%)	83/432 (19·2%)	27/298 (9·1%)	941/6593 (14·3%)
**Decrement in urinary QOL**							
<2× MCID	1193/1500 (79·5%)	··	941/1115 (84·4%)	721/933 (77·3%)	··	··	2855/3548 (80·5%)
≥2× MCID	307/1500 (20·5%)	··	174/1115 (15·6%)	212/933 (22·7%)	··	··	693/3548 (19·5%)
**Decrement in bowel QOL**							
<2× MCID	909/1498 (60·7%)	··	897/1111 (80·7%)	624/930 (67·1%)	··	··	2430/3539 (68·7%)
≥2× MCID	589/1498 (39·3%)	··	214/1111 (19·3%)	306/930 (32·9%)	··	··	1109/3539 (31·3%)

Data are n/N (%). CHHiP=Conventional or Hypofractionated High dose intensity modulated radiotherapy for Prostate cancer. FCCC=Fox Chase Cancer Center. GI=gastrointestinal. GU=genitourinary.

HYPRO=HYpofractionated irradiation for PROstate cancer. MCID=minimal clinically important difference. PROFIT=PROstate Fractionated Irradiation Trial. QOL=quality-of-life. RTOG=Radiation Therapy Oncology Group.

## Research in context

### Evidence before this study

We searched PubMed for peer-reviewed, original studies investigating the relationship between acute and late genitourinary and gastrointestinal toxicity following prostate radiotherapy from database inception up to Jan 1, 2024, using the following search criteria: “association” OR “relationship” AND “acute” AND “late” AND “toxicity” AND “prostate” AND “radiotherapy” OR “radiation therapy,” with no language restrictions. Key urological oncology and radiation oncology journals were also reviewed. We found scarce data from individual trials and single institution retrospective cohorts suggesting a potential association between physician-scored acute and late toxicity, predominantly in the setting of conventionally fractionated prostate radiotherapy. We did not find studies focusing on multiple prospective clinical trials, studies that clearly adjusted for differences in fraction size, dose, and radiation planning approach, or studies that robustly interrogated patient-reported late toxicity.

### Added value of this study

To our knowledge, this is the first individual patient data meta-analysis studying the association between acute and late toxicity following conventionally fractionated or moderately hypofractionated prostate radiotherapy that used data from multiple randomised clinical trials, allowing adjustment for key treatment variables and providing late toxicity in terms of both physician-scored and patient-reported metrics. We report that acute grade 2 or greater genitourinary and gastrointestinal toxicities were associated with late grade 2 or greater genitourinary and gastrointestinal toxicities as well as late decrement in urinary and bowel quality of life.

### Implications of all the available evidence

These data support the phenomenon of consequential late toxicity following radiotherapy in the context of prostate cancer and support efforts to evaluate whether interventions that reduce acute toxicity ultimately reduce the risk of late toxicity.
