# Supplementary material for: The interplay between acute and late toxicity among patients receiving prostate radiotherapy: an individual patient data meta-analysis of six randomised trials
Source: Lancet Oncol. Author manuscript; Available in PMC 2026 Feb 6. (PMC12875763; doi:10.1016/S1470-2045(24)00720-4)
Supplement: Supplementary [file NIHMS2080130-supplement-Supplementary.pdf]

THE LANCET
Oncology

Supplementary appendix

This appendix formed part of the original submission and has been peer reviewed. We post it as supplied by the authors.

Supplement to: Nikitas J, Jamshidian P, Tree AC, et al. The interplay between acute and late toxicity among patients receiving prostate radiotherapy: an individual patient data meta-analysis of six randomised trials. *Lancet Oncol* 2025; published online Jan 30. https://doi.org/10.1016/S1470-2045(24)00720-4.

Appendix

Supplementary Tables

Supplementary Table 1. Details of Radiotherapy Planning. ……………………………………*2*

Supplementary Table 2. Inter-Trial Heterogeneity Assessment.………………………………………*3*

Supplementary Table 3. Generalized linear mixed model analyzing the association between….4 acute grade ≥2 genitourinary (GU) or gastrointestinal (GI) toxicity and late grade ≥3 GU or GI toxicity.

Supplementary Table 4. Generalized linear mixed model analyzing the association between….5 acute grade ≥2 genitourinary (GU) or gastrointestinal (GI) toxicity and decrement at least twice the minimal clinically important difference (≥2x MCID) in patient-reported urinary or bowel quality-of-life (QOL) at 24 months.

Supplementary Table 5. Generalized linear mixed model analyzing the association between….6 acute grade ≥2 genitourinary (GU) or gastrointestinal (GI) toxicity and decrement at least twice the minimal clinically important difference (≥2x MCID) in patient-reported urinary or bowel quality-of-life (QOL) at the time of last available follow-up.

Supplementary Table 6. Generalized linear mixed model analyzing the association between….7 acute grade ≥2 genitourinary (GU) or gastrointestinal (GI) toxicity and decrement at least twice the minimal clinically important difference (≥2x MCID) in patient-reported urinary or bowel quality-of-life (QOL) using only patients that were not missing baseline QOL scores.

Supplementary Table 7. Predictive Performance of Generalized Linear Models for Late………8 Toxicity.

Supplementary Figures

Supplementary Figure 1. Forest plot showing the effect of acute grade ≥2 genitourinary……..9 (GU) toxicity on late ≥2 GU toxicity by trial.

Supplementary Figure 2. Forest plot showing the effect of acute grade ≥2 gastrointestinal……10 (GI) toxicity on late ≥2 GI toxicity by trial.

Supplementary Figure 3. Forest plot showing the effect of acute grade ≥2 genitourinary...……11 (GU) toxicity on decrement at least twice the minimal clinically important difference (≥2x MCID) for urinary quality-of-life (QOL).

Supplementary Figure 4. Forest plot showing the effect of acute grade ≥2 gastrointestinal……12 (GI) toxicity on decrement at least twice the minimal clinically important difference (≥2x MCID) for bowel quality-of-life (QOL).

Supplementary Table 1. Details of Radiotherapy Planning.

Trial	CTV	PTV Expansion	Planning Technique	IGRT
CHHiP	Low risk of SVI: Prostate + Proximal 2 cm of the SV<br><br>Intermediate/High risk of SVI: Prostate + Whole SV	1 cm (with reduction to 5-10 mm for sequential boost with IMRT)	3D-CRT: 58.4%<br>IMRT 30.0%	Confirmed IGRT: 28.4%
RTOG 0126	Prostate + Proximal 1 cm of SV	5-10 mm	3D-CRT: 66.2%<br>IMRT 33.8%	Weekly IGRT required
PROFIT	Low risk of SVI: Prostate<br><br>Medium/High risk of SV: Prostate + Proximal 1 cm SV	7 mm posteriorly, 10 mm other directions	Recommended IMRT, % not provided	Daily IGRT required
RTOG 0415	Prostate	4-10 mm	3D-CRT: 20.9%<br>IMRT: 79.1%	Daily IGRT required
Ottawa 0101	Prostate + Proximal 1 cm of SV	7 mm posteriorly, 10 mm other directions	3D-CRT	Weekly IGRT required
FCCC	Intermediate risk: Prostate + Proximal 9mm of the SV<br><br>High risk: Prostate + Whole SV + Pelvic nodes	5 mm posteriorly, 8 mm in all other directions (CF)<br><br>3 mm posteriorly, 7 mm in all other directions (MHF)	IMRT	Daily IGRT required

3D-CRT, 3-dimensional conformal radiation therapy; CF, conventional fractionation; CTV, clinical target volume; CHHiP, Conventional or hypofractionated high dose intensity modulated radiotherapy for prostate cancer; DRR, digitally reconstructed radiograph; FCCC, Fox Chase Cancer Center; IGRT, image-guided radiation therapy; IMRT, intensity-modulated radiation therapy; MHF, moderate hypofractionation; PROFIT, PROstate Fractionated Irradiation Trial; PTV, planning target volume; RTOG, Radiation Therapy Oncology Group; SV, seminal vesicle; SVI, seminal vesicle invasion.

Supplementary Table 2. Inter-Trial Heterogeneity Assessment.

**Acute Grade ≥2 GU Toxicity**			
**Endpoint**	**I^2**	**Cochran's Q**	***p*-value**
Late Grade ≥2 GU Toxicity	0%	2.42	0.79
Decrement ≥2x MCID in Urinary QOL	79%	9.54	0.0085
**Acute Grade ≥2 GI Toxicity**			
**Endpoint**	**I^2**	**Cochran's Q**	***p*-value**
Late Grade ≥2 GU Toxicity	0.4%	5.02	0.41
Decrement ≥2x MCID in Bowel QOL	0%	0.14	0.93

MCID, minimal clinically important difference; GI, gastrointestinal; GU, genitourinary; QOL, quality-of-life.

Supplementary Table 3. Generalized linear mixed model analyzing the association between acute grade ≥2 genitourinary (GU) or gastrointestinal (GI) toxicity and late grade ≥3 GU or GI toxicity.

**Late Grade ≥3 GU Toxicity**			
**Predictor**	**OR**	**95% CI**	***p*-value**
Acute Grade ≥2 GU Toxicity	2·16	1·58-2·97	<0·001
**Late Grade ≥3 GI Toxicity**			
**Predictor**	**OR**	**95% CI**	***p*-value**
Acute Grade ≥2 GI Toxicity	2·89	1·97-4·25	<0·001

Models were adjusted for age, androgen deprivation therapy (yes or no), type of radiation therapy (3-dimensional conformal radiation therapy or intensity-modulated radiation therapy), radiation dose (equivalent dose in 2 Gy fractions [EQD2] using $\alpha/\beta = 3$; <74 Gy, 74-80 Gy, or >80 Gy), and radiation schedule (conventionally fractionated or moderately hypofractionated). CI, confidence interval; OR, odds ratio.

Supplementary Table 4. Generalized linear mixed model analyzing the association between acute grade ≥2 genitourinary (GU) or gastrointestinal (GI) toxicity and decrement at least twice the minimal clinically important difference (≥2x MCID) in patient-reported urinary or bowel quality-of-life (QOL) at 24 months.

**Decrement ≥2x MCID in Urinary QOL at 24 Months**			
Predictor	OR	95% CI	*p*-value
Acute Grade ≥2 GU Toxicity	1·46	1·09-1·96	0·010
**Decrement ≥2x MCID in Bowel QOL at 24 Months**			
Predictor	OR	95% CI	*p*-value
Acute Grade ≥2 GI Toxicity	1·46	1·11-1·92	0·0069

Models were adjusted for age, androgen deprivation therapy (yes or no), type of radiation therapy (3-dimensional conformal radiation therapy or intensity-modulated radiation therapy), radiation dose (equivalent dose in 2 Gy fractions [EQD2] using $\alpha/\beta = 3$; <74 Gy, 74-80 Gy, or >80 Gy), and radiation schedule (conventionally fractionated or moderately hypofractionated). CI, confidence interval; OR, odds ratio.

Supplementary Table 5. Generalized linear mixed model analyzing the association between acute grade ≥2 genitourinary (GU) or gastrointestinal (GI) toxicity and decrement at least twice the minimal clinically important difference (≥2x MCID) in patient-reported urinary or bowel quality-of-life (QOL) at the time of last available follow-up.

**Decrement ≥2x MCID in Urinary QOL at Last Follow-Up**			
Predictor	OR	95% CI	*p*-value
Acute Grade ≥2 GU Toxicity	1·38	1·10-1·73	0·0056
**Decrement ≥2x MCID in Bowel QOL at Last Follow-Up**			
Predictor	OR	95% CI	*p*-value
Acute Grade ≥2 GI Toxicity	1·28	1·01-1·61	0·037

Models were adjusted for age, androgen deprivation therapy (yes or no), type of radiation therapy (3-dimensional conformal radiation therapy or intensity-modulated radiation therapy), radiation dose (equivalent dose in 2 Gy fractions [EQD2] using α/β = 3; <74 Gy, 74-80 Gy, or >80 Gy), and radiation schedule (conventionally fractionated or moderately hypofractionated). CI, confidence interval; OR, odds ratio.

Supplementary Table 6. Generalized linear mixed model analyzing the association between acute grade ≥2 genitourinary (GU) or gastrointestinal (GI) toxicity and decrement at least twice the minimal clinically important difference (≥2x MCID) in patient-reported urinary or bowel quality-of-life (QOL) using only patients that were not missing baseline QOL scores.

**Decrement ≥2x MCID in Urinary QOL**			
Predictor	OR	95% CI	*p*-value
Acute Grade ≥2 GU Toxicity	1·68	1·38-2·03	<0·001
**Decrement ≥2x MCID in Bowel QOL**			
Predictor	OR	95% CI	*p*-value
Acute Grade ≥2 GI Toxicity	1·67	1·31-2·14	<0·001

Models were adjusted for age, androgen deprivation therapy (yes or no), type of radiation therapy (3-dimensional conformal radiation therapy or intensity-modulated radiation therapy), radiation dose (equivalent dose in 2 Gy fractions [EQD2] using α/β = 3; <74 Gy, 74-80 Gy, or >80 Gy), and radiation schedule (conventionally fractionated or moderately hypofractionated). CI, confidence interval; OR, odds ratio.

Supplementary Table 7. Predictive Performance of Generalized Linear Models for Late Toxicity.

Endpoint	AUC	Sensitivity	Specificity
Late Grade ≥2 GU Toxicity	0.69	63%	63%
Late Grade ≥2 GI Toxicity	0.63	60%	60%
Decrement ≥2x MCID for Urinary QOL	0.57	56%	56%
Decrement ≥2x MCID for Bowel QOL	0.63	59%	59%

Using 5-fold cross-validation, we calculated the average area under the curve (AUC), sensitivity, and specificity for the generalized linear mixed models predicting late grade ≥2 genitourinary (GU) toxicity, late grade ≥2 gastrointestinal (GI) toxicity, decrement at least twice the minimal clinically important difference (≥2x MCID) for urinary quality-of-life (QOL), and decrement ≥2x MCID for bowel QOL.

Supplementary Figure 1. Forest plot showing the effect of acute grade ≥2 genitourinary (GU) toxicity on the odds ratio of late ≥2 GU toxicity by trial.

Trial	AcuteGU >=2 n/N	AcuteGU <2 n/N	OR (95% CI)	p-value
CHHIP	85/783	40/842	2.44 (1.65-3.65)	<0.001
RTOG0126	44/249	125/1212	1.73 (1.16-2.54)	0.0059
PROFIT	129/368	160/838	2.20 (1.63-2.97)	<0.001
RTOG0415	116/292	166/787	2.50 (1.85-3.38)	<0.001
OTT0101	16/71	39/361	2.34 (1.18-4.49)	0.012
FCCC	19/54	47/244	2.29 (1.16-4.43)	0.015
**Overall**	**409/1817**	**577/4284**	**2.20 (1.88-2.57)**	**<0.0001**

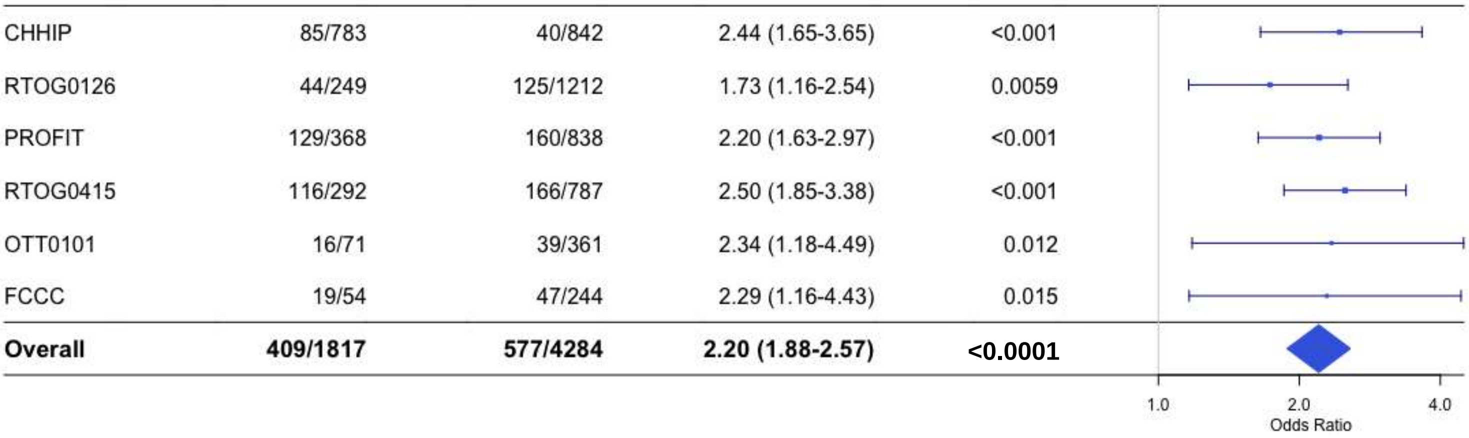

CHHiP, Conventional or hypofractionated high dose intensity modulated radiotherapy for prostate cancer; CI, confidence interval; FCCC, Fox Chase Cancer Center; GU, genitourinary; OR, odds ratio; PROFIT, PROstate Fractionated Irradiation Trial; RTOG, Radiation Therapy Oncology Group.

Supplementary Figure 2. Forest plot showing the effect of acute grade ≥2 gastrointestinal (GI) on the odds ratio of late ≥2 GI toxicity by trial.

	AcuteGI >=2	AcuteGI <2		
**Trial**	**n/N**	**n/N**	**OR (95% CI)**	**p-value**
CHHIP	93/500	88/1125	2.77 (2.01-3.82)	<0.001
RTOG0126	33/86	238/1375	2.76 (1.70-4.43)	<0.001
PROFIT	28/167	113/1039	1.80 (1.08-2.91)	0.020
RTOG0415	38/113	158/966	2.53 (1.61-3.92)	<0.001
OTT0101	7/21	76/411	2.53 (0.91-6.58)	0.061
FCCC	4/10	23/288	8.49 (1.90-35.46)	0.0033
**Overall**	**203/897**	**696/5204**	**2.53 (2.07-3.08)**	**<0.0001**

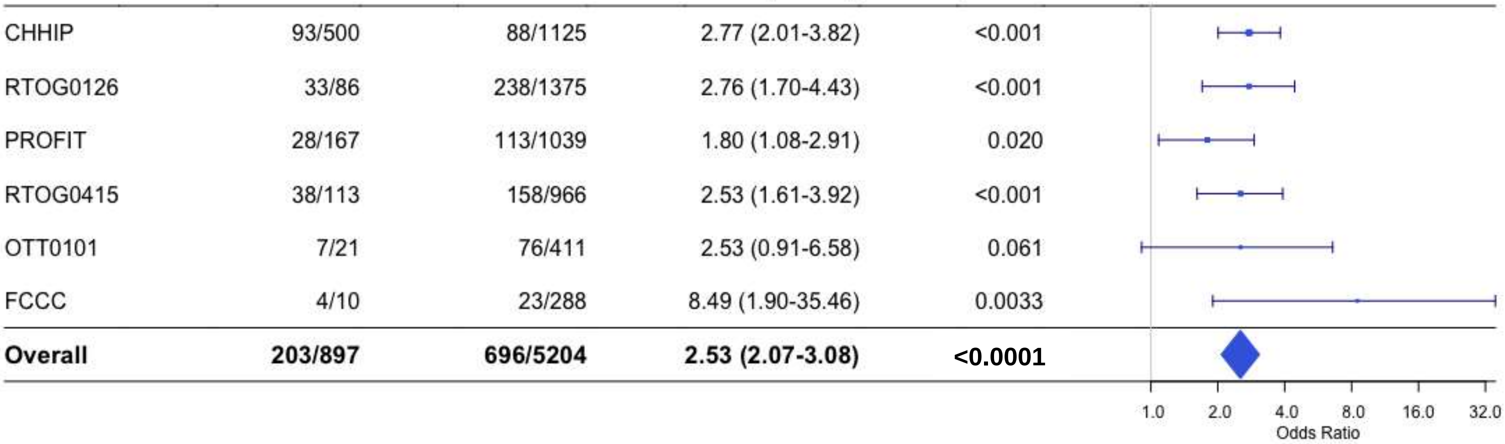

CHHiP, Conventional or hypofractionated high dose intensity modulated radiotherapy for prostate cancer; CI, confidence interval; FCCC, Fox Chase Cancer Center; GI, gastrointestinal; OR, odds ratio; PROFIT, PROstate Fractionated Irradiation Trial; RTOG, Radiation Therapy Oncology Group.

Supplementary Figure 3. Forest plot showing the effect of acute grade ≥2 genitourinary (GU) toxicity on the odds ratio of decrement at least twice the minimal clinically important difference (≥2x MCID) for urinary quality-of-life (QOL).

	AcuteGU >=2	AcuteGU <2		
**Trial**	**n/N**	**n/N**	**OR (95% CI)**	**p-value**
CHHIP	175/653	120/703	1.77 (1.36-2.30)	<0.001
PROFIT	69/344	105/771	1.57 (1.08-2.26)	0.016
RTOG0415	53/266	159/677	0.88 (0.61-1.26)	0.50
**Overall**	**297/1263**	**384/2151**	**1.41 (1.17-1.68)**	**0.0002**

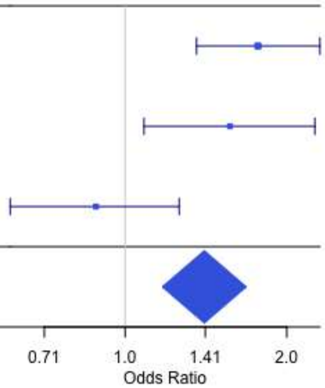

CHHiP, Conventional or hypofractionated high dose intensity modulated radiotherapy for prostate cancer; CI, confidence interval; GU, genitourinary; OR, odds ratio; PROFIT, PROstate Fractionated Irradiation Trial; RTOG, Radiation Therapy Oncology Group.

Supplementary Figure 4. Forest plot showing the effect of acute grade ≥2 gastrointestinal (GI) toxicity on the odds ratio of decrement at least twice the minimal clinically important difference (≥2x MCID) for bowel quality-of-life (QOL).

	AcuteGI >=2	AcuteGI <2		
Trial	n/N	n/N	OR (95% CI)	p-value
CHHIP	210/435	349/919	1.47 (1.16-2.30)	0.0014
PROFIT	43/155	171/956	1.58 (1.03-2.26)	0.033
RTOG0415	41/95	265/835	1.57 (1.01-1.26)	0.046
**Overall**	**294/685**	**785/2710**	**1.52 (1.26-1.83)**	**<0.0001**

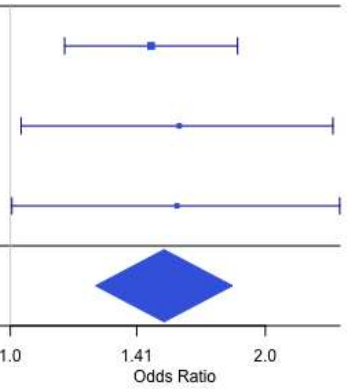

CHHiP, Conventional or hypofractionated high dose intensity modulated radiotherapy for prostate cancer; CI, confidence interval; GI, gastrointestinal; OR, odds ratio; PROFIT, PROstate Fractionated Irradiation Trial; RTOG, Radiation Therapy Oncology Group.
